# Quantifying sorptivity using the contact sponge method: an improved calculation method validated by classical capillary rise experiments and neutron radiography

**DOI:** 10.1617/s11527-026-03118-0

**Published:** 2026-05-16

**Authors:** H. Derluyn, J. Desarnaud, D. Vandevoorde, J. Dewanckele, M. N. Boone, S. Peetermans, E. Lehmann, H. Viles, V. Cnudde

**Affiliations:** 1 LFCR, Universite de Pau et des Pays de l’Adour, CNRS, Pau, France; 2 Institut Royal du Patrimoine Artistique, Koninklijk Instituut voor Kunst en Patrimonia, Parc du Cinquantenaire 1, 1000 Brussels, Belgium; 3 Rephine Stoneworks, Eeklostraat 148, 9030 Mariakerke, Belgium; 4 TESCAN XRE, Bollebergen, 2B, 9052 Ghent, Belgium; 5 Radiation Physics Research Group, Ghent University, Proeftuinstraat 86/N12, 9000 Gent, Belgium; 6 Tractebel Engineering, Boulevard Simon Bolivar 36, 1000 Brussels, Belgium; 7 Spallation Neutron Source Division, Paul Scherrer Institute, 5232 Villigen, Switzerland; 8 School of Geography and the Environment (SoGE), University of Oxford, S. Parks Road, Oxford, OX1 3QY United Kingdom; 9 Ghent University, PProGRess-UGCT, Krijgslaan 281/S8, 9000 Ghent, Belgium; 10 Environmental Hydrogeology, Utrecht University, Princetonlaan 8a, 3584 CB Utrecht, The Netherlands

**Keywords:** Porous stone, Water absorption, Contact-sponge method, Neutron imaging, Capillary absorption coefficient, Sorptivity

## Abstract

Non-destructive techniques such as the contact sponge method (CSM) and the Karsten tube have been developed to assess the water absorption properties of building materials during field measurements. In contrast, laboratory investigations typically rely on capillary rise tests (CR) on centimetric specimens imposing a one-dimensional water flow. Although the results obtained from these methods are qualitatively comparable, a robust quantitative correlation is still lacking. An analytical expression has previously been proposed to estimate the sorptivity from measurements with non-destructive techniques, based on the assumption that the water penetration depth equals the lateral spreading of water on the material surface. In this study, we investigate the water absorption processes induced by the contact sponge method, by combining neutron radiography, which allows direct visualization of water penetration, with conventional capillary rise tests based on gravimetric measurements. Sandstones and limestones with porosities ranging from 10% to 30% were examined. Neutron imaging reveals that the standard assumption of equal penetration depth and lateral spreading is not valid. These observations were used to propose an improved analytical expression that accounts for the three-dimensional water flow induced by the non-destructive method. This significantly improves the quantitative agreement between sorptivity values obtained from CSM and CR tests. This revised analytical framework results in an adaptation of the CSM field-testing protocol to ensure reliable sorptivity assessment.

**Supplementary Information:**

The online version contains supplementary material available at 10.1617/s11527-026-03118-0.

## Introduction

In the field of building stone conservation and sculpture restoration, non-destructive and in-situ techniques to measure materials properties are used the most, in order to maintain the integrity of heritage buildings and artefacts. Usually, in-situ measurements are applied to assess the efficiency of conservation treatments such as consolidants or water-repellent products [1, 2]. Mechanical properties can be determined with Drilling Resistance System measurements (semi-destructive test) [3, 4], the rebound test [5], or the peeling test [6]. The water content and water transport properties of building materials like stone and mortar are also essential parameters to measure because they play a crucial role in building material degradation [7–9]. Water has a crucial function in driving chemical and mechanical processes, as well as in promoting decay induced by biological colonisation [10, 11]. Salt, penetrating into a building material dissolved in water, is one of the most important causes of building material degradation. Variations in water content lead to cycles of crystallization and dissolution of salts which cause degradation [12–14]. Moreover, depending on the degree of water saturation, the presence of water will increase the risk of frost damage [15]. Consequently, the water content properties and water transport properties of building materials are of great interest to the field of building material conservation. Several non-destructive techniques enable in-situ measurements to assess the permeability and water absorption of porous building materials: Karsten tube (KT) [16, 17], Contact Sponge Method (CSM) [18–20] and the Mirowski pipe [20]. The Mirowski pipe is not in use anymore because of the difficulty of implementing it without leakage, leading to erroneous results. However, the KT and the CSM have complementary fields of application: KT provides more relevant results for higher absorption, whereas CSM is more suitable for stones with a lower absorption. These methods can be used to distinguish differences in pore structure, due to alterations or past treatments [21, 22], and to assess a conservation product by comparing results obtained before and after application. Although they are non-destructive, their drawbacks are that the results cannot be interpreted in terms of fundamental physical quantities. In order to obtain quantitative information, the Capillary Rise method (CR) is performed on stone samples in the laboratory [23, 24]. This method is destructive because several samples (5x5x5 cm^3^) have to be extracted from a building or monument for lab experiments.

Previous studies on the validation of measuring techniques for water absorption by porous materials have shown that the results of the different methods are related [22, 25]. They can be compared qualitatively, but there is no clear quantitative correlation, impairing adequate comparison of results of the different methods. Understanding the behaviour of water inside the complex pore network of building materials remains a crucial gap to providing a common calculation model. This paper investigates the water absorption processes induced by CSM inside stones, using neutron radiography imaging, and classical laboratory capillary rise measurements. Limestones as well as sandstones with a porosity ranging from 10% to 30% were tested. The visualized process was then compared to existing water absorption models in order to define an improved analytical expression for 3D flow of water from CSM for quantitative comparison with the results obtained with the capillary rise method. Finally, an easy calculation workflow for the use of practitioners in the field is given.

## Materials and methods

### Stones

Four limestones, Massangis Roche Jaune (MJ), Massangis Roche Claire (MC), Valanges (V) and Estaillades (E), and three sandstones, Stanton Moor (SM), Ohio Sandstone (OH) and Locharbriggs Sandstone (LB) were tested in this study. For the four limestone lithotypes, the open porosity and bulk density were determined on 16 samples (7x7x2 cm^3^) following standard EN 1936 [26]. For the three sandstone lithotypes, the open porosity and bulk density were determined on 5 samples (5x5x5 cm^3^) following the same standard. The values are reported in Table 1.

**Table 1:** Mean value and standard deviation of the bulk density and open porosity of the stone lithotypes used in this study

Lithotype	Symbol	Bulk Density (kgm^−3^)	Open porosity (%)
Massangis Roche Jaune	MJ	2400 ± 120	10.5 ± 0.9
Massagis Roche Claire	MC	2427 ± 5	10.7 ± 0.2
Valanges	V	2300 ± 100	14.0 ± 2.0
Estaillades	E	1920 ± 20	29.3 ± 6.0
Ohio Sandstone	OH	2084 ± 11	21.2 ± 0.2
Stanton Moor	SM	2323 ± 17	12.1 ± 0.7
Locharbriggs	LB	2070 ± 7	22.9 ± 1.3

### Water absorption methodologies

Two water absorption methodologies were employed: the classical capillary rise (CR) measurements, following EN 1925 [23], and the contact sponge method (CSM) according to UNI 11432-2011 [27]. Both techniques measure mass changes over time and provide a global assessment of the stones’ water absorption behaviour. However, they do not reveal how water is distributed internally. In the CR method, a homogenous stone sample is expected to exhibit a uniform horizontal water front advancing vertically from bottom to top, since the complete bottom surface of the stone is in contact with water. In contrast, the CSM involves a limited contact area, leading to a more complex three-dimensional waterfront as the water spreads spatially. To visualize this 3D spreading behaviour, neutron radiography was applied on four limestones: MJ, MC, V and E.

The CR and CSM experiments were performed on samples of 7 cm x 7 cm x 2 cm for the four limestone lithotypes. For the three sandstones, samples of around 10 cm x 10 cm in cross section, with a height between 4 and 7 cm, were used. The samples were dried in an oven at 60 ± 5 °C until a constant dry mass was reached. All laboratory measurements were performed at an average temperature of 20–22 °C and 55%±2% RH, using distilled water. Mass measurements were done with a balance with a precision of 0.001 g.

#### Capillary rise

**Fig. 1:**
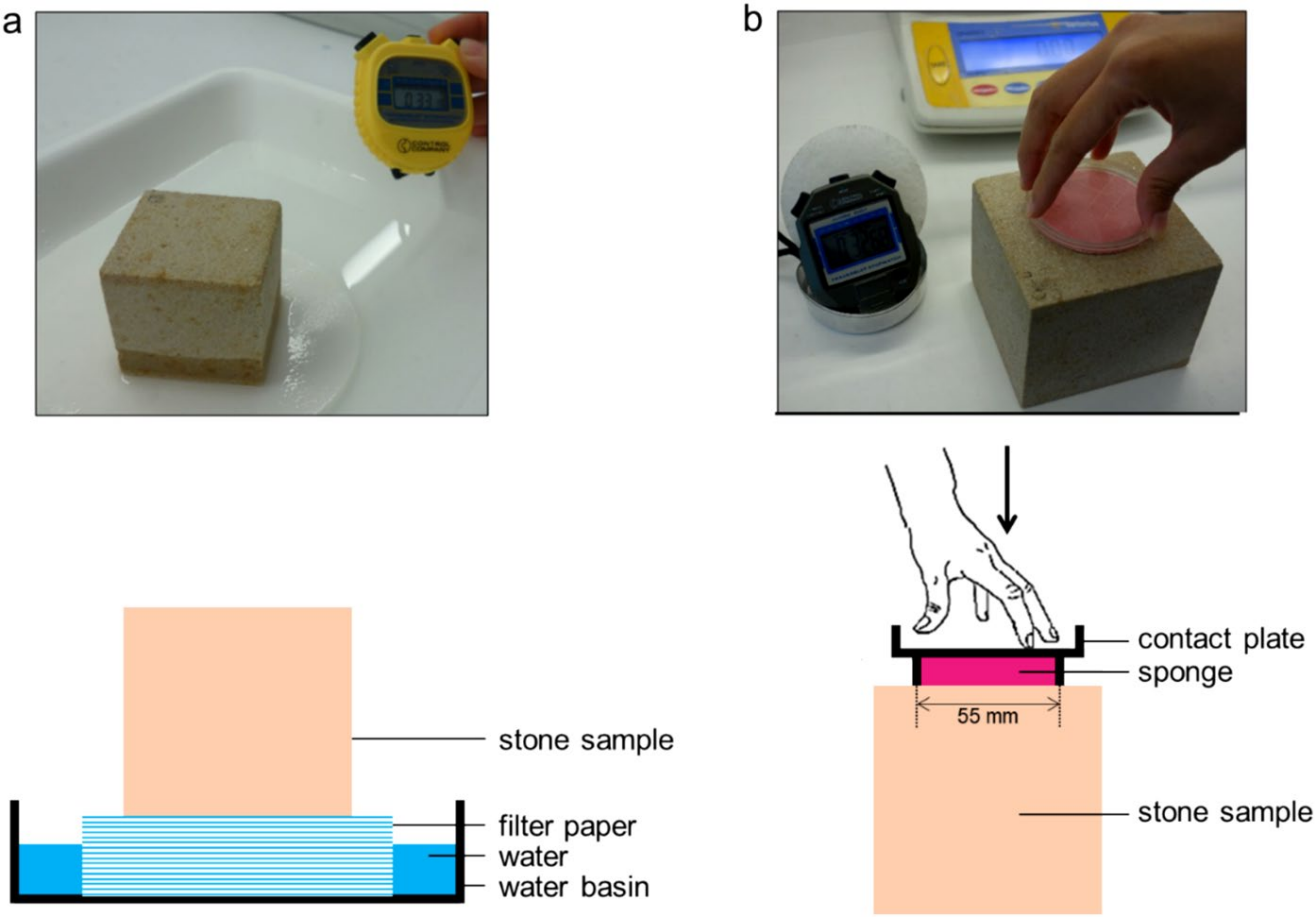
Illustration of the methods under study: **a** Capillary rise method, **b** Contact-sponge method (adapted from [22])

Stone samples (3 for each limestone, 5 for SM and OH, and 3 for LB) were placed on a pile of damp filter paper allowing the stone to absorb water upward by capillary forces (Fig. 1). The mass increase over time was monitored by measuring sample mass after fixed contact times by removing them from the filter paper, removing any water remaining on the surface with a damp cloth, and subsequently weighing the sample. When expressed as a function of the square root of time, this mass increase $\Delta m$ (kg) is typically linear during the initial stage of the capillary rise process. The cumulative inflow *Q* (kg/m^2^), the ratio between the mass increase and the contact surface exposed to water *A* (m^2^), is then defined as:

1 $$Q = \frac{\Delta m}{A} = A_{cap} \sqrt t = \frac{{\rho_{w} V_{abs} }}{A} = \rho_{w} S\sqrt t$$

Here, ${A}_{cap}$ is the capillary absorption coefficient (kg/m^2^s^1/2^), *t* the time, *ρ*_*w*_ is the density of water (kg.m^−3^), *V*_*abs*_ is the total absorbed volume of water (m^3^), and *S* is the sorptivity (m/s^1/2^). The capillary absorption coefficient is one of the basic transport properties for porous materials. Its equivalent when using a volumetric-based expression for the water uptake is the sorptivity *S = A*_*cap*_*/ρ*_*w*_.

**Fig. 2:**
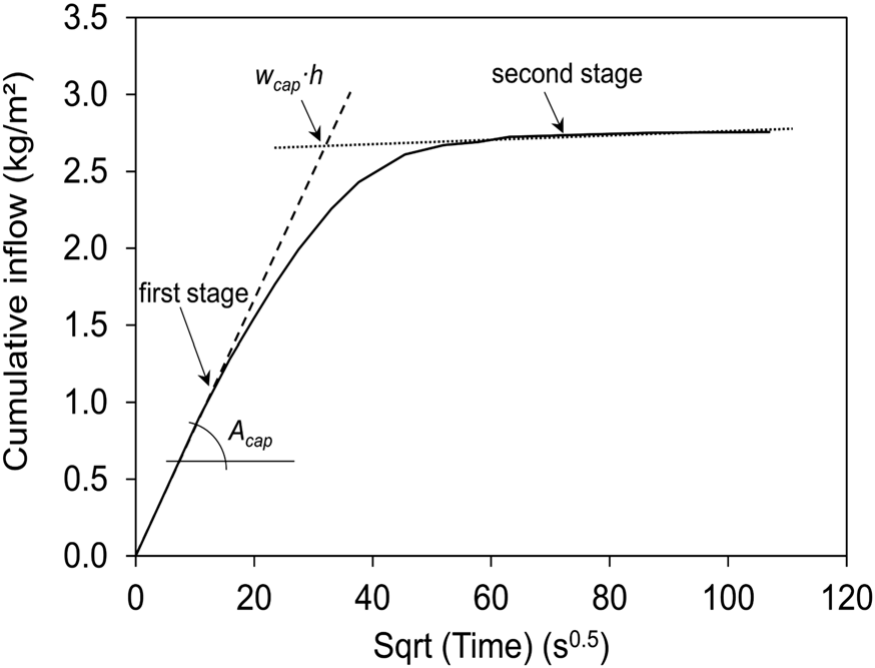
Capillary rise experiment represented as the cumulative water inflow versus square root of time. The slope of the first wetting stage defines the capillary absorption coefficient *A*_*cap*_, the intercept between the linear approximations of the first and second wetting stage defines the capillary water content *w*_*cap*_, with *h* the height of the sample

When measuring up to the point that the water reaches the top of the stone, the mass change will level off, and the intercept between the first linear uptake regime (slope ${A}_{cap}$ or slope *S*) and this second slower regime determines the capillary water content (*w*_*cap*_ (kg/m^3^) or *θ*_*cap*_=*w*_*cap*_/*ρ*_*w*_ (m^3^/m^3^)), as illustrated in Fig. 2.

#### Contact sponge method

The contact sponge method (CSM) [19, 20, 22] consists of a sponge enclosed in a contact box (Fig. 1b) composed of two parts, namely a base and a cover. Once the sponge is wet, the diameter of the sponge corresponds with the inner diameter of the base (5.5 cm), whereas the height of the sponge exceeds the vertical borders of the base. For the measurement, the cover of the contact plate is removed, water is added to the sponge and the sponge enclosed in the contact plate is weighed (*m*_*0*_). The cover of the contact plate is then removed and the sponge is pressed manually against the stone surface until the vertical borders of the base touch the stone surface. After the selected contact time, the contact sponge is removed and weighed inside the closed contact plate (*m*_*t*_).

The CSM was performed in the laboratory on 5 SM, 5 OH and 3 LB samples that were measured at 90, 120, 180, 240 and 300 seconds. The contact sponge was pressed from above against each sandstone sample. In addition, the diameter of the area wetted by the sponge at 300 seconds was measured on the contact surface.

In order to elucidate on the spreading of the water using the contact sponge method, neutron radiography was used to visualize CSM measurements on limestone samples of MC, MJ, V and E. For each stone type, two samples were tested.

#### Neutron radiography

Neutron radiography was performed at the NEUTRA beamline (NEUtron Transmission RAdiography) at the Paul Scherrer Institute in Villigen, Switzerland [28]. The samples were positioned between the neutron beam source and detector and time lapse radiographs with a time interval of 12.5 seconds were taken over a selected period of time depending on the stone type. The exposure time was 10 s per radiograph and the resulting radiographs have a spatial resolution of 100 µm/pixel. The wetted sponge was fixed to a remote-controlled elevator [29], allowing the sponge to be brought in contact with the middle of the bottom surface (7 cm x 7 cm) of the stone while acquiring neutron images (during which the operator needs to stay outside the experimental hutch for radiation protection). Neutrons penetrate through the stone sample and allow the acquisition of transmission images, i.e. radiographs, that include information on the internal composition of the samples. As neutrons interact strongly with hydrogen, a significant contrast is obtained between the wet and dry zones of the sample in the radiographs.

#### Image analysis

Image analysis of the time lapse data was implemented in LabView 2011 (National Instruments, Austin, TX, USA). A thresholding operation was performed to transform the gray value images into binarized images with two zones, i.e. wet and dry, where the interface represents the position of the waterfront. In the case of the multi-directional water front obtained with the contact sponge the horizontal width of the water front was determined as a function of the penetration depth for each binarized image, starting from the interface between the water supply and the stone. Assuming a cylindrical symmetry in the water volume, the wetted stone volume *V*_*wet*_ was determined by calculating the sum of the volumes of the cylinders with diameter equal to the horizontal width and with a height of one pixel (corresponding to 100 µm).

### Improved analytical expression for water flow and sorptivity using the contact sponge method

Equation 1 is only valid in case of a unidirectional absorption process [16, 30–34], which occurs when the contact area and the waterfront developed inside the stone remain equal, as is the case for CR. For the contact sponge method, imposing a restricted contact area with respect to the surface under investigation leads to an absorption process occurring in three directions. Assuming cylindrical symmetry, a 2D cross section of the water volume will show lateral spreading in addition to the ingress perpendicular to the contact surface. In the case of in-situ measurements, the only known variables are the total amount of absorbed water *∆m*, the radius of the contact area *r* and the width of the lateral spreading in function of time *x*_*0*_*(t)*. The question remains how the distribution of the volume of water can be calculated correctly from those data, and how sorptivity can be directly quantified based on a single in-situ test. Therefore, a more complex function than equation 1 is needed to express the absorbed amount of water as a function of time, and to deduce the sorptivity.

**Fig. 3:**
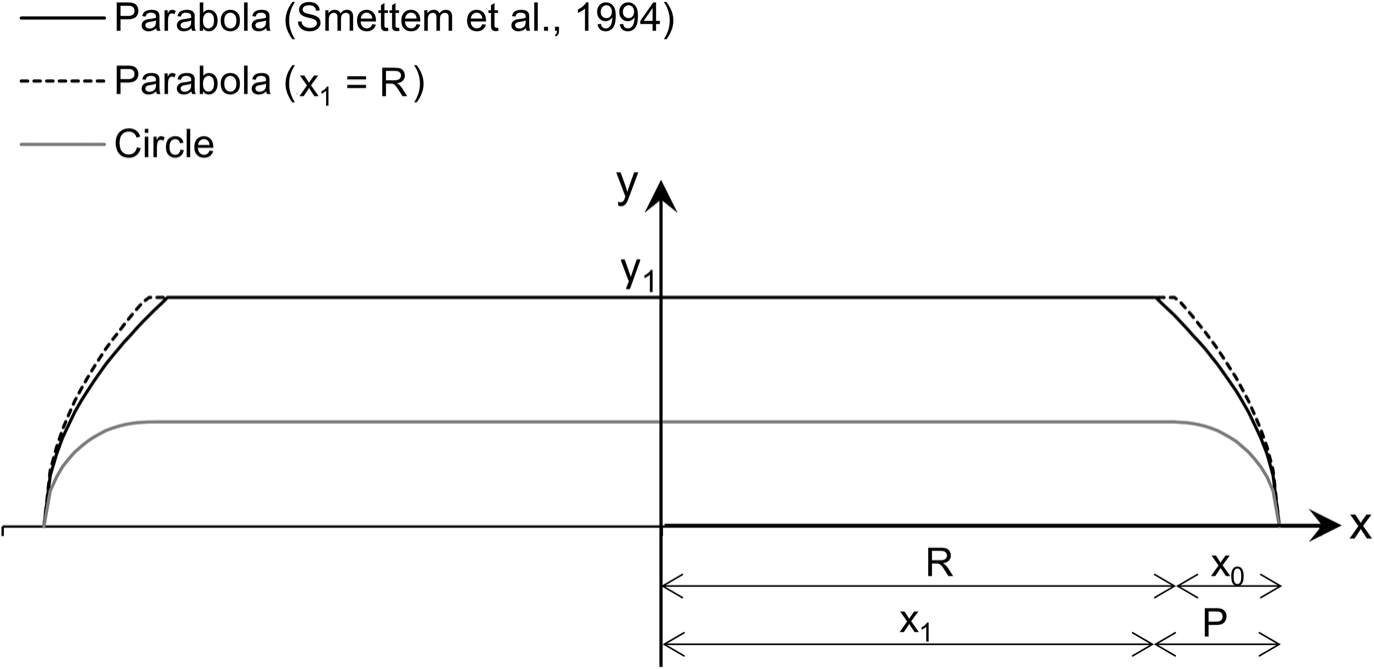
Schematic representation of the wetting front: *R* is the radius of the contact sponge and *x*_*0*_ is the lateral spreading of the wetting front. Considering the 2D part of the front to be parabolic (black line), *y*_*1*_ is the position of the 1D-front, (*x*_*1*_, *y*_*1*_) is the point where the 1D and 2D fronts meet, and *P* is the horizontal distance between this point and the vertex of the parabola (*R*+*x*_*0*_,0). Considering the 2D part of the front to be circular (grey line), the penetration depth of the 1D front equals *x*_*0*_

In the field of soil sciences, building on the work of Turner and Parlange (1974) [35], Smettem et al. (1994) [32] deduced an analytical formula for the three-dimensional flow out of a circular source using a sharp-front approximation, neglecting gravity. Considering a 2D cross section of the 3D volume, they describe the wetting front as being composed of a 1D horizontal front parallel to the water source and a 2D front that is parabolic (Fig. 3, black line). The following expression for the cumulative inflow of water is derived [32]:

2 $$V_{abs} = \pi R^{2} S\sqrt t + \frac{{\pi R\gamma S^{2} }}{{\theta_{cap} }}t$$

where* V*_*abs*_ (m^3^) is the total absorbed amount of water, *R* (m) is the radius of the circular source, *S* (m/s^1/2^) is the sorptivity, *θ*_*cap*_ (m^3^/m^3^) the capillary water content, *t* (s) the time and *γ* a constant, empirically determined to be equal to 0.75 by Smettem et al. (1994) [32]. Furthermore, they obtain the following relationships between the wetting front coordinates:

3 $$\frac{{x_{0} }}{{y_{1} }} = 0.456$$

4 $$\frac{{(R - x_{1} )}}{{y_{1} }} = 0.0913$$

Thus, by monitoring the lateral spreading *x*_*0*_, which can easily be measured on site, the penetration depth *y*_*1*_ as well as *x*_*1*_ can be calculated. Formula (3) implies that the penetration depth is 2.2 times larger than the lateral spreading of the water besides the radius of the cylindrical source.

The capillary water content ${\theta}_{cap}$ (m^3^/m^3^) is defined as the ratio between the (volumetric) absorbed amount of water (*V*_*abs*_ (m^3^)) and the total volume of stone saturated with water (*V*_*wet*_ (m^3^)):

5 $$\theta_{cap} = \frac{{V_{abs} }}{{V_{wet} }}$$

The wetted volume *V*_*wet*_ can then be calculated by considering this volume as a summation of cylinders over the penetration depth *y*_*1*_, with the radius of the cylinders changing as a function of *y*, defined by the parabola segment that borders the volume (as depicted in Fig. 3, black line). The parabola opens horizontally, with its vertex at (R+x_0_,0). The parabola crosses the point where the 1D and 2D wetting fronts meet (x_1_, y_1_), with the horizontal distance between this point and the vertex being equal to *P=R+x*_*0*_*-x*_*1*_. The expression for the wetted volume becomes (see detailed derivation in supplementary material S1):

6 $$V_{wet} = \pi y_{1} \left( {x_{1}^{2} + \frac{8}{15}P^{2} + \frac{4}{3}Px_{1} } \right)$$

Turner and Parlange (1974) [35] noted that *R-x*_*1*_ is much smaller than *y*_*1*_, and that it is doubtful that the difference between *R* and *x*_*1*_ can be measured with sufficient precision. If we consider, as a first approximation, *x*_*1*_ to be equal to the radius *R* of the circular source, the wetted volume can be rewritten as:

7 $$V_{wet} = \pi y_{1} \left( {R^{2} + \frac{8}{15}x_{0}^{2} + \frac{4}{3}x_{0} R} \right)$$

The values *x*_*0*_ and *y*_*1*_ can be easily obtained as a function of time from the neutron radiography series, where *x*_*0*_ is the lateral spreading measured from the border of the sponge, and *y*_*1*_ is the penetration depth of the absorbed volume at the position of the sponge’s border.

## Results and discussion

The average sorptivity and capillary water content values as obtained by capillary rise, together with their standard deviations, are listed in Table 2 for each lithotype.

**Table 2:** Overview of the mean sorptivity S and capillary water content θ_cap_ with their standard deviations for each stone type measured by CR

Lithotype	S (cm/min^0.5^)	θ_cap_ (m^3^/m^3^)
Massangis Roche Jaune	0.008 ± 0.002	0.046 ± 0.014
Massangis Roche Claire	0.014 ± 0.002	0.086 ± 0.004
Valanges	0.025 ± 0.003	0.116 ± 0.002
Estaillades	0.159 ± 0.007	0.220 ± 0.003
Ohio Sandstone	0.088 ± 0.043	0.135 ± 0.001
Stanton Moor	0.015 ± 0.003	0.079 ± 0.005
Locharbriggs	0.101 ± 0.013	0.165*

^*^This value was obtained from only one LB sample

**Fig. 4:**
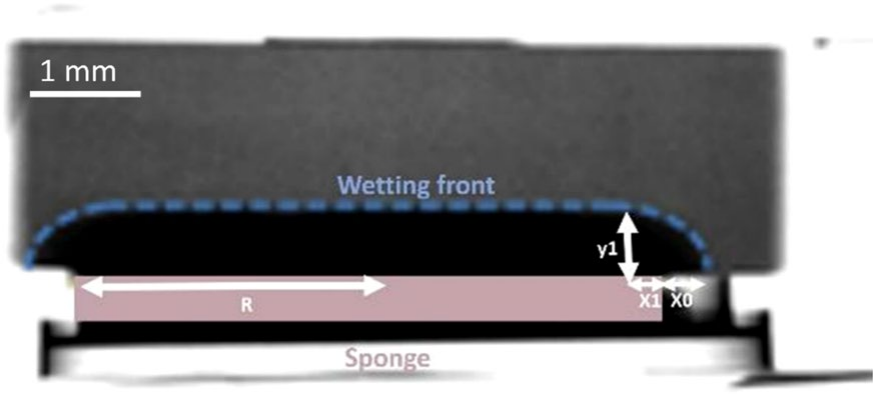
Neutron radiograph of the water absorption of a Valanges stone sample after 205 sec contact time with CSM

Neutron radiographs of the CSM reveal that the wetting front can be approximated by a combination of a horizontal and a curved component, as described in sect. 2.3 and illustrated in Fig. 4.

The neutron data corroborate the fact that the penetration depth *y*_*1*_ is different from the lateral spreading *x*_*0*_. Values for *y*_*1*_*/x*_*0*_ between 1.9 and 2.5 are found (corroborating the ratio predicted by Eq. (3)), with two outliers, a value of 1.3 for one MC sample, and a value of 5.3 for one MJ sample (we note here that the MJ stone has a heterogeneous character). The ratio *y*_*1*_*/x*_*0*_ remains quasi constant over the duration of the neutron acquisitions.

These findings differ from the assumption by Hendrickx (2012) [16], Wendler and Snethlage (1989) [30] and Vallet et al. [31] who worked on the Karsten tube method and assume that the penetration depth equals the lateral spreading *x*_*0*_, and thus, that the curved part of the wetting front is approximated by a circle. Using their assumption, the wetted volume *V*_*wet*_ (m^3^) equals (see detailed derivation in supplementary material S2, correcting for an error in [16]):

8 $$V_{wet} = \pi R^{2} x_{0} + \frac{2}{3}\pi \left( {x_{0}^{3} + \frac{3}{4}\pi Rx_{0}^{2} } \right)$$

**Fig. 5:**
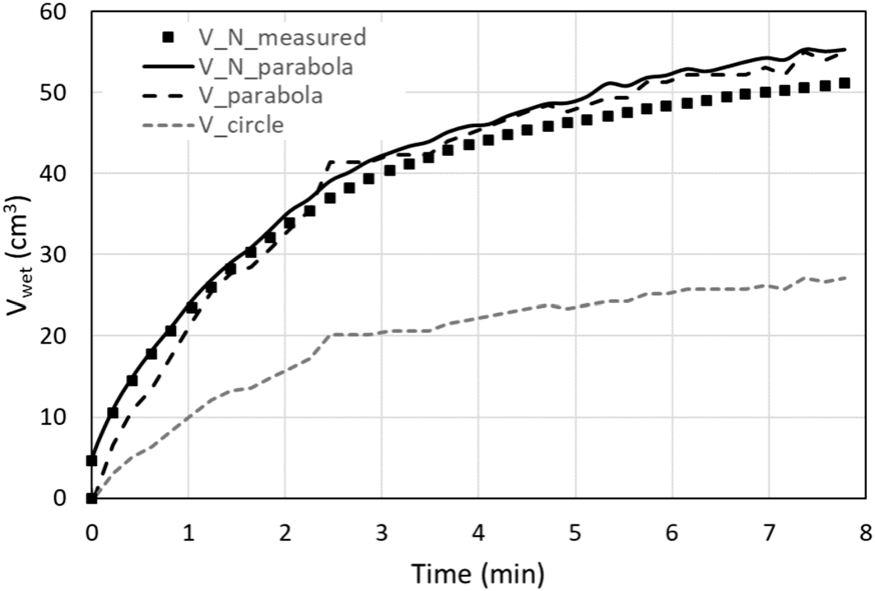
The measured wetted volume of Estaillades limestone, together with calculated volumes using different analytical expressions (see text for details)

The wetted volume obtained from the neutron radiography image analysis is presented in Fig. 5 for an Estaillades limestone sample (V_N_measured), together with the volume calculated through Eq. (7) using the measured *x*_*0*_ and *y*_*1*_ distances (V_N_parabola). In addition, the volume calculated through Eq. (6) using the scaling laws (3) and (4), so uniquely based on the measurement of *x*_*0*_, is plotted as V_parabola, and the volume calculated through Eq. (8) assuming *y*_*1*_ equals *x*_*0*_ is plotted as V_circle.

Similar graphs are obtained for all other limestone samples. These results confirm that the assumption of *x*_*0*_=*y*_*1*_ strongly underestimates the wetted volume, and that the approximation with a parabolic shape for the curved part of the wetting front reflects the real wetted volume much more closely. When the penetration depth is unknown, it can be calculated using Eq. (3), and the volume results from Eq. (6) using Eqs. (3) and (4).

**Fig. 6:**
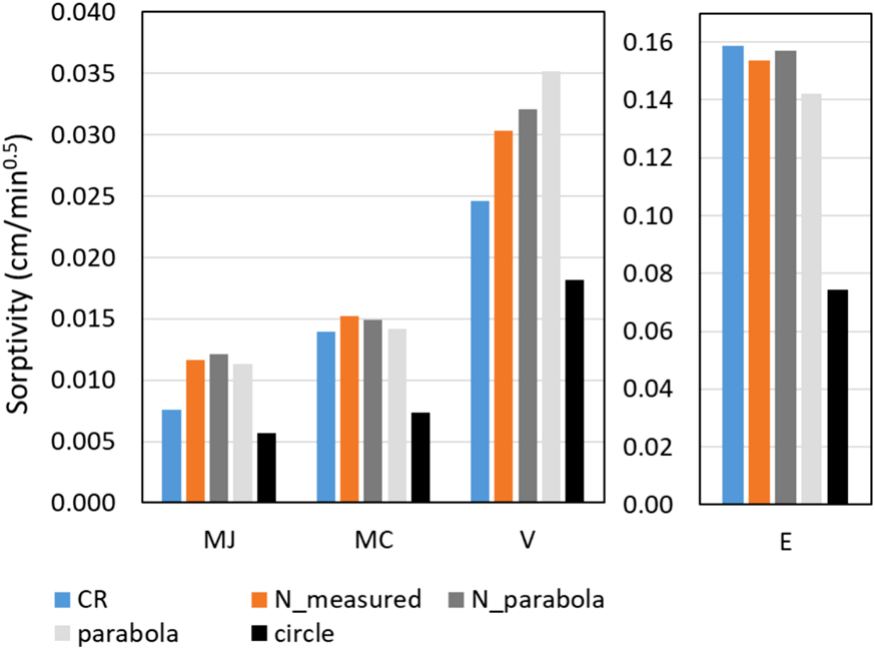
The sorptivity values for each limestone, obtained from classical capillary rise (CR, blue), and from CSM experiments as measured by neutron radiography (N_measured, orange) and approximated by different analytical descriptions: using the measured *x*_*0*_ and *y*_*1*_ distances from neutron radiographs (N_Parabola, dark gray), using the *x*_*0*_ distance and Smettem’s scaling laws (parabola, light gray), and assuming a quarter circle for the curved component of the wetting front (circle, black)

We subsequently determined the sorptivity of the limestone samples by calculating the absorbed volume as $\theta_{cap} V_{wet}$ using the capillary water content reported in Table 1, and determining the best fit for Eq. (2). The sorptivity values for each limestone, calculated using the parabolic analytical descriptions (gray values) are presented in Fig. 6, and can be compared to the value obtained from the classical capillary rise measurements (represented in blue). The results show that sorptivity values in the same range as the CR-values are retrieved, and that there is only a small difference between sorptivity calculated from the volumes derived from the neutron radiography analysis and calculated from the analytical descriptions using measured *x*_*0*_ and *y*_*1*_ distances (dark gray values), and using the *x*_*0*_ distance combined with the scaling proposed by Smettem et al. (1994), Eqs. (3) and (4) (light gray values). However, the assumption that the penetration depth equals the lateral spreading leads to a systematic underestimation of the sorptivity (black values).

The results obtained above provide us with a methodology for deducing the sorptivity from CSM tests performed on site, with the prerequisite that the lateral spreading, *x*_*0*_, is being measured.

Using this methodology, the sorptivity S and capillary water content *θ*_*cap*_ of three sandstones (SM, OH and LB) were estimated. The diameter *D* of the area wetted by the sponge at 300 seconds was measured on the contact surface in centimetres:

9 $$x_{0} = D/2 - 2.75$$

where 2.75 is the radius of the sponge in centimetres. The following steps were performed to determine the sorptivity:

1. *V*_*wet*_ was calculated at 300 seconds using the measured *x*_*0*_-value in equation (6), employing the scaling laws (3) and (4);
2. *θ*_*cap*_ was calculated using Eq. (5) with ${V}_{abs}=\Delta m/{\rho}_{w}$ the absorbed amount of water deduced from the mass change of the contact sponge measured at 300 seconds, and *V*_*wet*_ from step 1;
3. the sorptivity results from finding the best fit of Eq. (2) on the CSM data, using *θ*_*cap*_ calculated in step 2.

**Fig. 7:**
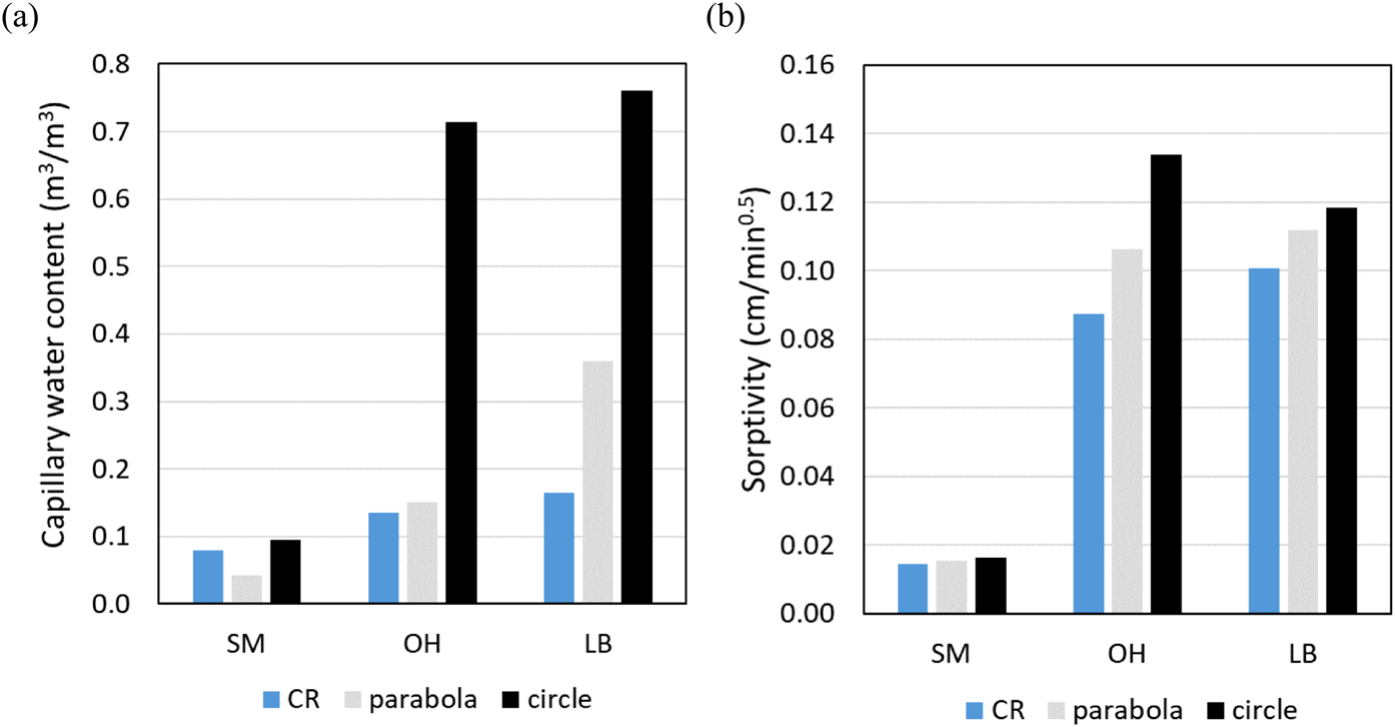
Capillary water content *θ*_*cap*_ **a** and sorptivity *S* **b** for SM, OH and LB sandstone obtained with CR and from the CSM using the calculation method based on a parabolic-curved wetting front (light gray) and based on a circular-curved wetting front (black)

The *θ*_*cap*_ and sorptivity values resulting from this calculation are given in Fig. 7 (light gray values). To compare with the more straightforward assumption that the penetration depth equals the lateral spreading *x*_*0*_, the calculation was repeated by using in step 1 the Eq. (8) instead of (6). The resulting values are represented in black. For completeness and comparison, the values obtained from CR are represented in blue in the figure as well.

The results show that the approximation of a circular-curved wetting front overestimates the sorptivity and the capillary water content *θ*_*cap*_ , for this last one up to values that are physically not possible (e.g. for OH sandstone, the *θ*_*cap*_-value of 0.71 would require a porosity of more than 71%, and for LB sandstone a porosity of more than 76%). On the other hand, the *θ*_*cap*_-values found with the approximation of a parabolic-curved wetting front are physically possible, and the sorptivity values are closer to the ones obtained from the CR tests.

## Conclusions and recommendations

We have directly imaged the water penetration during the CSM test for different stones by means of neutron radiography, showing that the standard assumption that the penetration depth of the moisture front equals the lateral spreading of water on the material surface is not valid. This permitted us to refine the analytical expression for the water uptake during the CSM, and to retrieve sorptivity values that are much more in line with the ones found during a classical capillary rise test. Our experimental campaign included limestones and sandstones with porosities ranging from 10 to 30%; and sorptivities ranging between 0.01 and 0.16 cm/min^0.5^. Future research could focus on extending the experiments to other types of stones or building materials, covering a larger range of porosity and sorptivity values.

Our improved calculation thus provides more accurate results for the quantification of sorptivity by non-destructive in-situ CSM testing. It requires an adaptation of the field test protocol for the CSM: once the test is finished (that is, after 5 minutes), just after the sponge is removed from the stone surface for weighing, the diameter of the wetted area, *D*, has to be measured immediately. This diameter corresponds to the lateral spreading of the water inside the substrate. If the stain is not completely circular, it is important to measure the diameter at several positions and to average them out. From the diameter measured, the lateral spreading, *x*_*0*_, can be calculated by using equation 9. Once *x*_*0*_ is known, the corresponding wetted volume can be calculated from Eq. (6); the needed parameters *y*_*1*_ and *x*_*1*_ defined by the laws (3) and (4). The capillary water content, *θ*_*cap*_, is then calculated from equation 5, with the absorbed volume of water defined by the mass change of the sponge after 5 minutes. The final sorptivity *S* is deduced from the results obtained from the contact sponge measurement by fitting the equation (2) on the data points. An Excel file including an example and a spreadsheet for use during field measurement campaigns is provided as supplementary material.

## Supplementary Information

Below is the link to the electronic supplementary material.

Supplementary file1 (XLSX 21 kb)

Supplementary file2 (DOCX 20 kb)

## Data Availability

Data will be made available on reasonable request.
