# Supplementary material for: Quantifying sorptivity using the contact sponge method: an improved calculation method validated by classical capillary rise experiments and neutron radiography
Source: Mater Struct. 2026 May 16;59(5):235. doi: 10.1617/s11527-026-03118-0 (PMC13179236; doi:10.1617/s11527-026-03118-0)
Supplement: Supplementary file 2 — Supplementary file2 (DOCX 20 kb) [file 11527_2026_3118_MOESM2_ESM.docx]

Supplementary material

S1. Analytical expression for the wetted volume bordered by a parabola

Looking at Figure 3, the volume is determined by the rotation around the y-axis of the surface defined by the parabola, as pointed out in the papers by Turner and Parlange (1974) [35] and Smettem et al. (1994) [32].

The parabola passes the points (*R+x_0_*,*0*) and (*x_1_*,*y_1_*), where *R* is the radius of the contact area, *y_1_* the penetration depth (in the *y*-direction), and *x_0_* the additional spreading of the wetting front in the *x*-direction with respect to the sponge’s radius. The horizontal distance between (*x_1_*,*y_1_*) and the vertex equals *P=R+x_0_-x_1_*. The axis of symmetry of the parabola is the *x*-axis, and the parabola is generally described by:

$x=ay^{2}+by+c$ (S1.1)

where the vertex (*R+x_0_*,*0*) equals $\left( \frac{4ac-b^{2}}{4a},-\frac{b}{2a} \right)$, thus *b*=0 and *c*=*R*+*x_0_=x_1_+P.* Knowing that the parabola passes (*x_1_*,*y_1_*) leads to $a=-\frac{P}{{y1}^{2}}$ and the parabola is defined by:

$x=-\frac{P}{{y_{1}}^{2}}y^{2}+P+x_{1}$ (S1.2)

The horizontal cross section of the wetted volume is a disk with radius *x* and the volume over an infinitesimal height *dy* equals:

$\pi x^{2}dy$ (S1.3)

By integrating over the height *y_1_*, the total wetted volume becomes:

$V_{wet}=\pi\int_{0}^{y_{1}} \left( -\frac{P}{{y_{1}}^{2}}y^{2}+P+x_{1} \right)^{2}dy$ (S1.4)

$V_{wet}=\pi\int_{0}^{y1} \left( \frac{P^{2}}{{y_{1}}^{4}}y^{4}+\left( P+x_{1} \right)^{2}-2\frac{P}{{y_{1}}^{2}}y^{2}\left( P+x_{1} \right) \right)^{2}dy$

$V_{wet}=\pi\left[ \frac{P^{2}}{{y_{1}}^{4}}\frac{y^{5}}{5}+{y\left( P+x_{1} \right)}^{2}-2\frac{P}{{y_{1}}^{2}}\frac{y^{3}}{3}\left( P+x_{1} \right) \right]_{0}^{y1}$

$V_{wet}=\pi\left( P^{2}\frac{y_{1}}{5}+{y_{1}\left( P+x_{1} \right)}^{2}-2P\frac{y_{1}}{3}\left( P+x_{1} \right) \right)$

$V_{wet}=\pi\left( P^{2}\frac{y_{1}}{5}+y_{1}P^{2}+y_{1}{x_{1}}^{2}+2Px_{1}y_{1}-2P\frac{y_{1}}{3}x_{1}-2P^{2}\frac{y_{1}}{3} \right)$

$V_{wet}=\pi\left( y_{1}{x_{1}}^{2}+y_{1}P^{2}\left( \frac{1}{5}+1-\frac{2}{3} \right)+2Px_{1}y_{1}\left( 1-\frac{1}{3} \right) \right)$

$V_{wet}=\pi\left( y_{1}{x_{1}}^{2}+\frac{8}{15}y_{1}P^{2}+\frac{4}{3}Px_{1}y_{1} \right)$

$V_{wet}=\pi y_{1}\left( {x_{1}}^{2}+\frac{8}{15}P^{2}+\frac{4}{3}Px_{1} \right)$ (S1.5)

In the special case where $x_{1}$*=R* and *P* thus equals *x_0_*, the expression for the volume becomes:

$V_{wet}=\pi y_{1}\left( R^{2}+\frac{8}{15}{x_{0}}^{2}+\frac{4}{3}x_{0}R \right)$ (S1.6)

S2. Analytical expression for the wetted volume bordered by a circle

Following the idea presented by Hendrickx [16], the wetted volume *V_wet_* consists of the volume of a cylinder *V_cyl_* and the volume of a ring surrounding the cylinder *V_ring_*:

$V_{wet}=V_{cyl}+V_{ring}$ (S2.1)

The volume of the cylinder is defined as:

$V_{cyl}=\pi R^{2}T$ (S2.2)

with *R* the radius of the contact area, and *T* the penetration depth.

The volume of the ring can be calculated by summing up the annular areas of the horizontal cross sections of the ring over the ring’s height. The area of an annulus is calculated by subtracting two circles, an outer circle with radius $R+\sqrt{T^{2}-y^{2}}$ and an inner circle with radius *R*. The volume of a ring slice is then given by:

$V_{ring slice}= \pi\left( \left( R+\sqrt{T^{2}-y^{2}} \right)^{2}-R^{2} \right)dy$ (S2.3)

Rewriting equation (S2.3) results in:

$V_{ring slice}= \pi\left( T^{2}-y^{2}+2R\sqrt{T^{2}-y^{2}} \right)dy$ (S2.4)
(Note that this equation is different from equation (2) in Hendrickx’ paper [16], which contains an error)

The ring volume is then calculated as the integral:

$V_{ring}=\int_{0}^{T} \pi\left( T^{2}-y^{2}+2R\sqrt{T^{2}-y^{2}} \right)dy$ (S2.5)

Knowing that the integral of a function of the type $\sqrt{T^{2}-y^{2}}$ is given by:

$\int\sqrt{T^{2}-y^{2}}dy=\frac{1}{2}y\sqrt{T^{2}-y^{2}}+\frac{1}{2}T^{2}\tan^{-1} \frac{y}{\sqrt{T^{2}-y^{2}}}+cte$ (S2.6)

the ring volume is found by evaluating:

$V_{ring}=\left[ \pi\left( T^{2}y-\frac{y^{3}}{3}+Ry\sqrt{T^{2}-y^{2}}+RT^{2}\tan^{-1} \frac{y}{\sqrt{T^{2}-y^{2}}} \right) \right]_{0}^{T}$ (S2.7)

and results in:

$V_{ring}=\pi\left( T^{3}-\frac{T^{3}}{3}+RT^{2}\frac{\pi}{2} \right)=\frac{2}{3}\pi\left( T^{3}+\frac{3}{4}\pi RT^{2} \right)$ (S2.8)

(Note again the difference with equation (3) in Hendrickx’ paper [16])

Combining equations (S2.2) and (S2.7), the total wetted volume becomes:

$V_{wet}= \pi R^{2}T+\frac{2}{3}\pi\left( T^{3}+\frac{3}{4}\pi RT^{2} \right)$ (S2.9)

Equation (S2.8) holds when assuming that the ring volume is described by the rotation of a quarter circle around the cylinder, thus the penetration depth *T* equals the spreading of the wetting front in the *x*-direction *x_0_*.
